# Myeloid-derived suppressor cells—new and exciting players in lung cancer

**DOI:** 10.1186/s13045-020-0843-1

**Published:** 2020-01-31

**Authors:** Zhenzhen Yang, Jiacheng Guo, Lanling Weng, Wenxue Tang, Shuiling Jin, Wang Ma

**Affiliations:** 1 Department of Oncology, The First Affiliated Hospital of Zhengzhou University, NO.1 Eastern Jianshe Road, Zhengzhou, 450052 Henan China; 2 Department of Cardiology, The First Affiliated Hospital of Zhengzhou University, Zhengzhou, 450052 Henan China; 3 Henan Province Key Laboratory of Cardiac Injury and Repair, Zhengzhou, 450052 Henan China; 4 Departments of Otolaryngology, The Second Affiliated Hospital of Zhengzhou University, Zhengzhou, 450000 Henan China; 5 Center for Precision Medicine of Zhengzhou University, NO.40 North Daxue Road, Zhengzhou, 450052 Henan China

**Keywords:** Lung cancer, MDSCs, Immunosuppression, Anticancer

## Abstract

Lung cancer (LC) is the leading cause of cancer-related death worldwide due to its late diagnosis and poor outcomes. As has been found for other types of tumors, there is increasing evidence that myeloid-derived suppressor cells (MDSCs) play important roles in the promotion and progression of LC. Here, we briefly introduce the definition of MDSCs and their immunosuppressive functions. We next specifically discuss the multiple roles of MDSCs in the lung tumor microenvironment, including those in tumor growth and progression mediated by inhibiting antitumor immunity, and the associations of MDSCs with a poor prognosis and increased resistance to chemotherapy and immunotherapy. Finally, we also discuss preclinical and clinical treatment strategies targeting MDSCs, which may have the potential to enhance the efficacy of immunotherapy.

## Introduction

Within the global cancer burden, lung cancer (LC) is the most commonly diagnosed cancer, accounting for 11.6% of all cancer cases, and the leading cause of cancer-related death, accounting for 18.4% of all cancer-related deaths [1]. Despite tremendous advances in LC treatment, the overall survival (OS) of LC patients remains poor. The tumor immune microenvironment (TIME) is dynamic during malignant progression, in which all cells in the TIME are involved, especially T lymphocytes; these cells are now considered to be key components of the autoanticancer response and have been shown to have therapeutic effects in a large proportion of cases by blocking “immune checkpoint” activation. However, despite the increasing success of these new therapies, a large number of patients have not benefited. Therefore, there is an urgent need to identify new elements of the host immune system that can serve as relevant biomarkers and therapeutic targets. Recent studies have increasingly emphasized the role of the TIME in the treatment of resistance. Immunosuppressive cells, such as bone marrow-derived suppressor cells (MDSCs), tumor-associated macrophages (TAMs), and regulatory T (Treg) cells, act as suppressive TIME components to attenuate immune responses [2]. Among these cells, MDSCs have roles in the prognosis, development, and treatment of LC that have been paid increasing attention [3, 4]. MDSCs are a group of highly heterogeneous cells derived from immature myeloid progenitors that are usually divided into two subpopulations: polymorphonuclear MDSCs (PMN-MDSCs) and monocytic MDSCs (M-MDSCs) [5–7]. MDSCs, as revealed by current research, are involved not only in the control of antitumor immune responses but also in tumor progression by promoting tumor angiogenesis, tumor cell invasion, and premetastatic niche formation [8, 9]. The levels of MDSCs are also closely related to clinical outcomes and therapeutic effects in LC patients [10]. In this review, we discuss the major subtypes and inhibitory functions of MDSCs in LC, as well as their clinical significance. In addition, we also emphasize that MDSC-targeted therapeutic procedures, administered either alone or in combination with chemotherapeutic and/or immunotherapeutic regimens, have potential therapeutic benefits in LC.

## Main phenotypic and functional characteristics of MDSCs

Early studies in mice have highlighted a shared, systemic expansion of myeloid cells bearing the markers CD11b and Gr1, but current research suggests that MDSCs can be divided into two large groups of cells termed PMN-MDSCs (CD11b^+^Ly6G^+^Ly6C^lo^), which are phenotypically and morphologically similar to neutrophils, and M-MDSCs (CD11b^+^Ly6G^−^Ly6C^hi^), which are similar to monocytes in terms of morphology and phenotype [6, 10, 11]. In humans, according to morphological and phenotypic features, the cells equivalent to PMN-MDSCs are mostly defined as CD11b^+^CD14^−^CD15^+^/CD66b^+^ cells, while M-MDSCs can be defined as CD11b^+^CD15^−^CD14^+^HLA-DR^−/low^ cells [12]. In addition, M-MDSCs also express the myeloid marker CD33, and PMN-MDSCs display CD33^dim^ staining [13]. However, Lin^−^ (including CD3, CD14, CD15, CD19, CD56) HLA-DR^−/low^CD33^+^ cells are groups of MDSCs comprising relatively immature progenitors. These immature cells are identified as eMDSCs [6]. However, there are still no uniform standards for surface molecular markers of human MDSCs, and many other markers are being reported gradually. From the perspective of LC, according to multicolor immunofluorescence staining evaluated by fluorescence-activated cell sorting (FACS), the following phenotypes of MDSCs have been reported (shown in Table 1): CD11b^+^CD14^−^CD15^+^CD33^+^ [14], CD11b^+^CD14^+^S100A9^+^ [15], CD16^low^CD11b^+^CD14^−^HLA-DR^−^CD15^+^CD33^+^ [16], CD14^+^HLA-DR^−/low^ [17, 18], B7-H3^+^CD14^+^HLA-DR^−/low^ [19], CD11b^+^CD14^−^HLA-DR^−^CD33^+^CD15^+^ILT3^high^ [20], Lin^−^CD14^−^CD11b^+^CD39^+^CD73^+^ [21], Lin^−^CD14^+^CD11b^+^CD39^+^CD73^+^ [21], Lin^−^CD14^+^CD15^+^H LA-DR ^−^[22], Lin^−^CD14^+^CD15^−^HLA-DR ^−^[22], Lin^−^CD14^−^HLA-DR ^−^[22], Lin-CD33^+^CD14^+^CD15^−^HLA-DR ^−^[25], CD33^+^CD11b^+^CD14 ^−^[27], CD33^+^CD11b^+^CD14^+^HLA-DR^−/low^ [27], and CCR5^+^HLA-DR^−/low^CD11b^+^CD14^+^CD15 ^−^[28].

**Table 1:** Clinical significance of MDSCs in lung cancer

Refs.	Phenotype (MDSCs)	Tumor tissue (TT)/ peripheral blood (PB)	NSCLC/SCLC	No. of patients	Implications
[14]	CD11b^+^CD14^−^CD15^+^CD33^+^	PB	Advanced NSCLC	41	Decreased in the advanced-stage patients who had clinical benefit (PR or SD) and in the early-stage patients after removal of tumor.
[15]	CD11b^+^CD14^+^S100A9^+^	PB	Advanced NSCLC	24	Poor chemotherapy response and short PFS
[16]	CD16^low^CD11b^+^CD14^−^HLA-DR^−^CD15^+^CD33^+^	PB	Advanced NSCLC	185	Significantly increased compared to healthy controls
[17]	CD14^+^HLA-DR^−/low^	PB	NSCLC	60	Negatively correlated with PFS
[18]	CD14^+^HLA-DR^−/low^	PB	SCLC	42	Independent biomarker for poor prognosis
[19]	B7-H3^+^CD14^+^HLA-DR^−/low^	PB	NSCLC	111	Decreased RFS
[20]	CD11b^+^CD14^−^HLA-DR^−^CD33^+^CD15^+^ ILT3^high^	PB	Stage IV NSCLC	105	Decreased OS
[21]	lin^−^CD14^−^CD11b^+^ CD39^+^/CD73^+^PMN-MDSCs lin^−^CD14^+^CD11b^+^ CD39^+^/CD73^+^M-MDSCs	PB	NSCLC	24	Decreased with chemotherapy cycles in SD and PR groups, increased in PD group.
[22]	Lin^−^CD14^ + ^CD15^ + ^CD11b^ +^ CD33 ^+ ^HLA-DR^−^	PB	NSCLC	110	Independent prognostic marker for decreased PFS and OS.
[23]	Lin^−^CD14^−^HLA-DR^−^	PB	NSCLC	46	After three cycles, bevacizumab-based chemotherapy significantly reduced the level of Lin^−^CD14^−^HLA-DR^−^ cells.
[24]	Lox-1^+^ PMN-MDSCs	PB	NSCLC	34	Patients with a higher ratio of Tregs to Lox-1^+^PMN-MDSCs in the blood after the 1st nivolumab had better PFS.
[25]	Lin^−^CD33 ^+^ CD14^ +^ CD15^−^ HLA-DR^−^	PB	Metastatic NSCLC	61	Decreased OS in anti-PD-1 treatment.
[26]	SSC^low^Lin^−^HLA-DR^−/LOW^CD33^+^ CD13^+^CD11b^+^CD15^+^CD14^−^	PB	stage IIIB or IV NSCLC	53	PMN-MDSCs (≥6 cell/μl) showed a significantly improved survival in anti-PD-1 treatment.
[27]	CD33^+^CD11b^+^CD14^−^ PMN-MDSCs CD33^+^CD11b^+^CD14^+^HLA-DR^−/low^ M-MDSCs	PB	NSCLC	7	Both subtypes decreased after SBRT treatment.
[28]	CD11b^+^HLA-DR^−/low^CD14^−^CD15^+^ PMN-MDSCs CCR5^+^HLA-DR^−/low^CD11b^+^CD14^+^CD15^−^ M-MDSCs	TT and PB	Resectable NSCLC	42	TT PMN-MDSCs displayed higher PD-L1 expression levels than the same cells in the PB. Significant correlations between lower total PMN-MDSCs and CCR5^+^ M-MDSCs frequencies in the peripheral blood and improved RFS.

*PR* partial response, *SD* stable disease, *PD* progressive disease, *PFS* progress free survival, *RFS* recurrence-free survival, *OS* overall survival, *SBRT* stereotactic body radiotherapy

The criteria for characterizing the phenotype of MDSCs by flow cytometry are relatively defined, and immunosuppressive function is a functional standard defined for MDSCs. While MDSCs were initially described as merely T cell suppressive, emerging evidence suggests that MDSCs also interact with and modulate the function of other immune cells, particularly macrophages (Mø) [29, 30], NK cells [31, 32], Treg cells [33], and B cells [34]. Moreover, MDSCs, TAMs, and dendritic cells (DCs) have been reported to interact and cross-promote their immunosuppressive activities in the tumor microenvironment [35]. Most of the available data indicate that MDSCs have different functional characteristics between the peripheral lymphoid organs and tumor tissues [36]. In most reports, the proportion of PMN-MDSCs in the peripheral lymphoid organs is much higher than that of M-MDSCs. Moreover, PMN-MDSCs have relatively moderate suppressive activity and play a major role in the regulation of tumor-specific immune responses, ultimately leading to the development of tumor-specific T cell tolerance. In tumor tissue, MDSCs have relatively strong suppressive functions, and M-MDSCs account for a greater proportion and more suppression than PMN-MDSCs and can rapidly differentiate into TAMs and DCs [37]. These findings suggest that targeting only one branch of myeloid cells (monocytes and/or Mø or granulocytes) or only intratumoral populations will not be sufficient for achieving therapeutic benefits. They may also indicate that the differences in the mechanisms regulating MDSC function in tumors and the peripheral lymphoid organs affect targeted therapies directed at these cells.

## Mechanisms underlying MDSC-mediated immunosuppression in LC

**Fig. 1:**
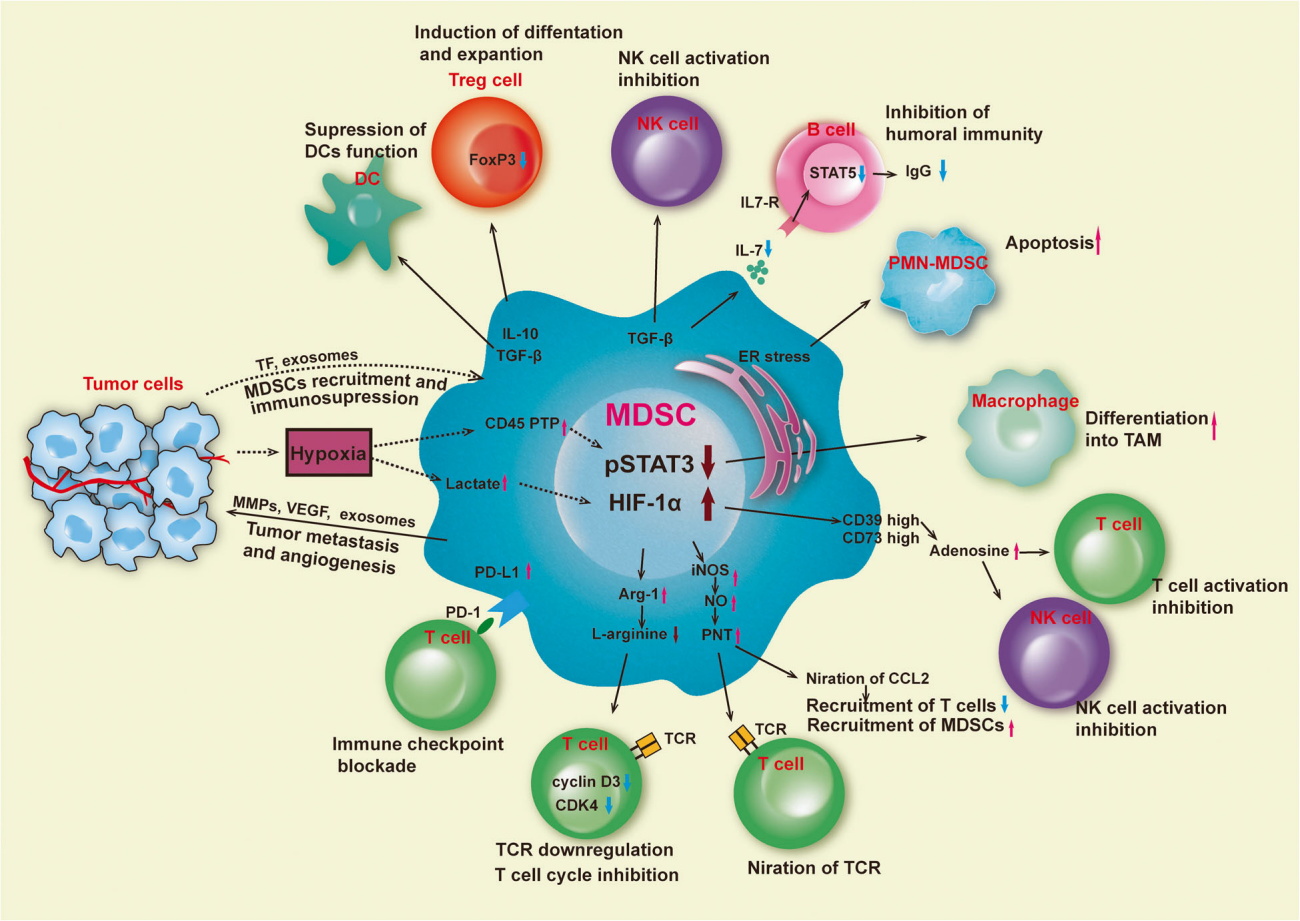
Immunosuppressive functions of MDSCs in the tumor microenvironment. DCs: dendritic cells; TAM: tumor-associated macrophage; ER: endoplasmic reticulum; Arg-1: arginase 1; iNOS: inducible nitric oxide synthase; HIF-1α: hypoxia-inducible factor-1α; STAT3: signal transducer and activator of transcription 3; VEGF: vascular endothelial growth factor; TF: tissue factor. In the tumor microenvironment, MDSCs are exposed to hypoxic conditions. This leads to an increase in HIF-1α-mediated elevation of Arg1 and iNOS and upregulation of inhibitory PD-L1 on the MDSC surface, all of which can suppress T cell immune activity. It also produces IL-10 and TGF-β, etc., which attract Treg cells to the tumor site and enhance their immunosuppressive functions, while suppressing the functions of B cells, NK cells, and DCs. Adenosine from CD39-high/CD73-high MDSCs is a further major NK suppressive factor. Much of the STAT3 activity in MDSCs is greatly reduced due to the effects of hypoxia. This leads to the rapid differentiation of M-MDSCs to TAMs. PMN-MDSCs die quickly due to ER stress. Factors released by dying cells can promote immunosuppressive mechanisms. At the same time, MDSCs can promote tumor angiogenesis and metastasis by producing VEGF, MMPs, and exosomes. Tumor tissue-derived exosomes can also affect MDSC recruitment and immunosuppression

MDSCs are the major suppressor population of the immune system, with the ability to inhibit adaptive and innate immune responses. The immunosuppressive mechanisms of MDSCs have been elucidated, especially in cancer growth, since MDSCs play a key role in tumor evasion of immune surveillance (Fig. 1).

### Metabolic mechanisms

Metabolic reprogramming is a core requirement for tumor cells to meet the energy needs of rapid cell proliferation and to adapt to the tumor microenvironment. This event leads to altered cellular signaling, enzymatic activity, and/or metabolic flux during disease, such as the initiation of aerobic glycolysis (Warburg effect) and changes in oxidative phosphorylation, which can penetrate the tumor microenvironment and affect immune cells [38].

MDSCs that inhibit T cell function mainly depend on the following three metabolic modes: (1) Arginase (Arg)-1 consuming arginine, (2) inducible nitric oxide synthase (iNOS) producing nitric oxide (NO), and (3) processes producing reactive oxygen species (ROS), including the superoxide anion (O2–), hydrogen peroxide (H2O2), and peroxynitrite (PNT) (ONOO–). The inhibitory activity of Arg-1 is based on its role in the hepatic urea cycle, which metabolizes l-arginine into l-ornithine. Increased accumulation of Arg-1 results in l-arginine depletion from the microenvironment, an event that inhibits T cell proliferation by reducing T cell CD3δ expression [14, 39] or by preventing T cells from upregulating the cell expression of the cycle regulators cyclin D3 and Cyclin-dependent kinase 4 (CDK4), thereby arresting the cell cycle in the G0/G1 phase [40]. A pegylated form of the catabolic enzyme Arg I (peg-Arg I) can enhance the growth of tumors in mice in a manner that correlates with increased MDSC numbers through general control of the nonrepressed-2 eIF2α kinase [41]. NO can react with superoxide to form PNT and then directly inhibit T cells by nitrifying TCR, thereby reducing the affinity of TCR for antigen-MHC complexes presented by cancer cells and blocking the migration of T cells by nitrating T cell-specific chemokines [42, 43]. The two major subpopulations of MDSCs exploit different mechanisms to inhibit T cell proliferation. PMN-MDSCs express high levels of ROS and low levels of NO, whereas M-MDSCs are the opposite, and both subpopulations express arginase. Given that ROS are unstable and active only for a short period of time, PMN-MDSCs require tight cell-cell contact to act on T cells. This contact is provided by antigen-specific interactions with T cells [44]. M-MDSCs produce large amounts of NO, Arg1, and immunosuppressive cytokines. These molecules have a much longer half-life than ROS and require cell access, but no close contact is required between M-MDSCs and T cells. Therefore, M-MDSCs are mainly effective in inhibiting nonspecific T cell responses [7, 36].

In a preclinical model of pancreatic cancer, silencing lactate dehydrogenase isoform A decreased the frequency of MDSCs in the mouse spleen and resulted in improved cytolytic function of NK cells. Accordingly, lactate, which induces hypoxia-inducible factor-1α (HIF-1α), acts as an endogenous inhibitor of histone deacetylases (HDACs), regulating the transcription of genes involved in metabolism and immunity [45] and thereby increasing the frequency of MDSCs generated from mouse bone marrow cells in vitro [46]. The increase in CD45 tyrosine phosphatase activity in the hypoxic environment of the tumor promotes the downregulation of STAT3 expression, thereby promoting the differentiation of M-MDSCs into TAMs [30].

The ATP: AMP ratio of cells, as an integral part of overall metabolism, is monitored by mTOR. mTOR-mediated HIF-1α induction plays an important role in activating glycolysis [47]. Ectonucleotidases are localized on tumor cells and different populations of immune cells that hydrolyze ATP or ADP into AMP via CD39 (ectonucleoside triphosphate diphosphohydrolase-1) and further process AMP into adenosine via CD73 (ecto-5′-nucleotidase) in the tumor microenvironment [48, 49]. Li et al. recognized that TGF-β-induced HIF-1α activation was mTOR dependent in MDSCs in non-small cell lung cancer (NSCLC) patients and was critical for CD39/CD73 induction under normoxic conditions. Metformin inhibited the expression and activity of CD39/CD73 through activation of AMPKα and inhibition of the HIF-1α pathway to impair MDSC immunosuppressive activity, thereby improving antitumor T cell responses [50].

### STAT signaling pathway

One of the major factors that drives MDSC expansion in cancer is transcriptional factor signal transducer and activator of transcription 3 (STAT3). The activation of STAT3 can inhibit apoptosis in myeloid cells and prevent these cells from differentiating into mature cells by promoting the expression of the antiapoptotic genes Bcl-xL, c-myc, and cyclin D1 [51]. Spontaneous bronchoalveolar adenocarcinoma in CCSP-rtTA/(tetO)7-Stat3C bitransgenic mice is directly induced by the overexpression of constitutively active Stat3C in alveolar type II (AT II) epithelial cells and continued activation of the Stat3 signaling pathway [52]. Wu et al. showed that the overexpression of Stat3C in ATII epithelial cells induced the accumulation of MDSCs in the lungs and blood of transgenic mice and that phosphorylation of the proto-oncogenic intracellular signaling molecules Stat3, Erk1/2, and P38 was significantly increased in MDSCs, accompanied by decreases in the frequencies of CD4^+^ and CD8^+^ T cells. In the bronchioalveolar lavage fluid and plasma of doxycycline-treated transgenic mice, the concentrations of the MDSC-associated cytokines IL-1β, IL-6, IL-10, IL-13, INF-γ, TNF-α, and GM-CSF were significantly increased, which stimulated alveolar monocytes/macrophages to undergo MDSC conversion in vitro [53]. However, a recent study showed that STAT3 had a new role in the differentiation of MDSCs independent of HIF-1α. The authors reported profound downregulation of STAT3 activity in MDSCs in tumors compared to the same cells in the peripheral lymphoid organs and blood. This reduction was a key factor in regulating the differentiation of MDSCs into TAMs. Hypoxia caused an increase in CD45 tyrosine phosphatase activity in M-MDSCs, which resulted in a selective decrease in STAT3 activity in M-MDSCs in tumors. Upregulation of CD45 phosphatase activity was mediated by a disruption in CD45 protein dimerization caused by increased CD45 sialylation [30].

Wang et al. revealed that B cell differentiation and function were impaired during tumor progression in mouse models of LC. Most of the impairments were attributable to the direct effects of MDSCs, and they were related to and seemingly mediated by a IL-7-mediated reduction in STAT5 signaling and dysfunctional B cell responses. In addition, the authors revealed that TGF-β (an MDSC-associated cytokine) was a central inhibitor of IL-7 signaling in B cells in the TIME [34].

### Exosomes

A growing number of studies have demonstrated that MDSC-released exosomes have a role in immunosuppression [54, 55]. Deng et al. found that although doxorubicin treatment was largely efficacious in inhibiting primary tumors, it significantly increased the incidence and burden of pulmonary metastasis through miR-126a^+^ MDSC-derived exosomes. Induction of miR-126a^+^ MDSC-derived exosomes by doxorubicin strongly increased the expression of the inflammatory cytokine IL-13. These proinflammatory changes promoted the outgrowth of both MDSCs and Th2 cells in the lungs, where these cells increased angiogenesis and promoted the lung metastasis of breast cancer via miR-126a^+^ MDSC-derived exosomes [56]. Interestingly, exosomes from tumor cells also contribute to the function of MDSCs. A study found that the expression of PD-L1 in MDSCs could be increased after tumor-derived exosomes were transferred from tumor cells to MDSCs in glioma and LC tumor models. This expression was related to the increased expression of Arg1 in MDSCs, the production of TGF-β, and the strengthened immunosuppressive activity of these cells [57].

### Caspase recruitment domain-containing protein 9

Caspase recruitment domain-containing protein 9 (CARD9) is mainly expressed in myeloid cells [58, 59] and is an important adaptor protein in innate immunity, as it regulates innate immunity and affects adaptive immune responses [60, 61]. Qu et al. demonstrated the role of CARD9 in the development of LC for the first time. They found that CARD9 prevented LC development by suppressing the expansion of MDSCs and production of indoleamine 2,3-dioxygenase (IDO). Further studies suggested that knocking down CARD9 expression enhanced the expression of IDO in MDSCs through the noncanonical NF-κB pathway, which manifested as increased expression of NIK, p-P100, p52, and RelB. Thus, the CARD9-NF-κB-IDO pathway in MDSCs can inhibit the suppressive function of MDSCs and prevent LC development [62].

### Long noncoding RNAs

Long noncoding (Lnc) RNAs are highly important factors associated with tumors and may be used as markers for tumor diagnosis, which is valuable for targeted therapy.

The lncRNA HOXA transcript antisense RNA myeloid-specific 1 (HOTAIRM1), which is transcribed by RNA polymerase II, is located between the human HOXA1 and HOXA2 genes. HOTAIRM1/HOXA1 has been shown to modulate myeloid cell differentiation [63]. Knocking down HOTAIRM1 expression significantly inhibits the expression of HOXA1 and HOXA4 and reduces the induction of myeloid differentiation gene transcripts, such as CD11b and CD18, during all-trans retinoic acid (ATRA)-induced granulocytic differentiation [64]. In the tumor tissue of LC patients, Tian et al. found that HOTAIRM1 was expressed in CD11b^+^CD33^+^HLA-DR^−^CD14^−^ MDSCs, while overexpression of HOTAIRM1 downregulated the expression of Arg-1 and ROS in MDSCs. In different LC subtypes, HOTAIRM1 was mainly expressed in MDSCs in lung adenocarcinoma. The specific mechanism may be that HOTAIRM1 can enhance the expression of HOXA1 in MDSCs and high levels of HOXA1 can delay tumor progression and enhance the antitumor immune response by downregulating the immunosuppression mediated by MDSCs [65]. The lncRNA metastasis-associated lung adenocarcinoma transcript 1 (MALAT1), also known as nuclear enrichment autosomal transcript 2 (NEAT2), is widely expressed in mammals [66, 67]. Abnormal expression of MALAT1 is observed in a variety of tumor tissues and is associated with a number of important biological processes, including proliferation, apoptosis, invasion, and metastasis [68, 69]. Zhou et al. showed that the relative expression of the lncRNA MALAT1 in peripheral blood mononuclear cells (PBMCs) from LC patients was significantly lower than that in PBMCs from healthy controls. The relative expression of MALAT1 was moderately negatively correlated with the proportion of CD33^+^CD11b^+^HLA-DR^−^ MDSCs. In vitro results indicated that knocking down MALAT1 expression significantly increased the proportion of MDSCs [70]. Almost simultaneously, their team discovered another lncRNA regulated by MDSCs. They showed that the lncRNA RUNXOR exhibited upregulated expression in MDSCs isolated from the peripheral blood of LC patients and that the expression of RUNX1, a target gene of RUNXOR, was downregulated. Furthermore, the expression of RUNXOR was higher in MDSCs isolated from tumor tissues than in cells from adjacent tissues. After RUNXOR knockdown, the expression of Arg1 in MDSCs was downregulated, and RUNX1 expression was restored. These results indicated that RUNXOR might affect the function of MDSCs by regulating RUNX1 [71]. Zheng et al. first reported that HIF-1α upregulated the expression of the lncRNA Pvt1 in PMN-MDSCs under hypoxic conditions. Pvt1 played a key role in regulating the immunosuppressive capacity of PMN-MDSCs. Pvt1 knockdown reduced the levels of Arg1 and ROS in PMN-MDSCs and restored antitumor T cell responses. Therefore, the concept of targeting Pvt1 to attenuate PMN-MDSC-mediated immunosuppression may be a potential therapeutic strategy [72].

### MicroRNA

MicroRNA (miRNA) networks regulate the differentiation, expansion, and suppression function of MDSC in the tumor microenvironment through different signaling pathways [73]. Research by Li et al. revealed that in LLC tumor models, miR-155 and miR-21 can promote the proliferation and immunosuppressive function of MDSCs via targeting SHIP-1 and phosphatase and tensin homolog, respectively, leading to STAT3 activation [74]. Subsequently, Wang et al. reported that miR-155 deficiency promotes solid tumor growth through increasing the recruitment of MDSCs to the tumor microenvironment and enhancing the tumor-promoting functions of the recruited MDSCs [75]. In addition, miR-9 regulates MDSC differentiation by targeting runt-related transcription factor 1, an essential transcription factor in the regulation of MDSC differentiation and function [76].

### Tissue factor

Exogenous coagulation, tissue factor (TF), and its noncoagulant isoforms have been found to promote both tumor progression and poor clinical outcomes [77, 78]. In a xenograft model, TF knockdown in A549 cells resulted in a significant decrease in fibrin deposition, with smaller concomitant reductions in C3b/iC3b/C3c and C5b-9 deposition, which represented complement activation, with relatively few MDSCs infiltrates and inhibited tumor growth [79]. These findings suggest that in the tumor microenvironment, TF-induced coagulation activates the complement system and subsequently recruits MDSCs to promote tumor growth.

### Endoplasmic reticulum stress

The endoplasmic reticulum (ER) stress response is an evolutionarily conserved mechanism for protecting cells from various stress conditions, including hypoxia, nutrient deficiencies, and low pH. In the tumor microenvironment, ER stress is associated with the fate of MDSCs. Condamine and colleagues found that in tumor-bearing mouse models of lung cancer, thymoma, colon cancer, and breast cancer, PMN-MDSCs induced more apoptosis than neutrophils or monocytes. Further studies have shown that this effect is mediated by changes in TNF-related apoptosis-induced ligand receptor (TRAIL-R) expression induced by ER stress in MDSCs. The short survival of MDSCs stimulates the proliferation of their precursors and further triggers their expansion [80].

In addition to mediating immunosuppressive mechanisms, MDSCs also affect the remodeling of the tumor microenvironment and tumor angiogenesis by producing VEGF and MMP9 [56], thereby promoting tumor progression. Interestingly, it has been reported that MDSCs can regulate the biology of cancer stem cells by affecting the IL-6/STAT3 and NO/NOTCH signaling pathways [81]. MDSCs endow stem-like qualities to multiple myeloma cells by inducing piRNA-823 expression and DNMT3B activation [82]. However, there is currently no published study on whether MDSCs affect the stemness of LC cells.

## Significance of MDSCs in LC

As previously stated, various factors affecting MDSC expansion have been studied in multiple types of cancers and across various biological models. Like many other cancers, LC has high levels of MDSCs that are associated with resistance to chemotherapy, targeted therapy, and immunotherapy and can predict a poor prognosis [14–17, 70] (Table 1).

### Role of MDSCs in the development of LC

A study of 185 Caucasian patients with NSCLC found that the frequency of CD16^low^CD11b^+^CD14^−^HLA-DR^−^CD15^+^CD33^+^ PMN-MDSCs was significantly increased in the NSCLC patients compared with healthy controls [16]. Liu et al. identified MDSCs with a CD11b^+^CD14^−^CD15^+^CD33^+^ phenotype in treatment-naive advanced NSCLC patients. These cells expressed IL-4R, IFN-γR, Arg-1, and iNOS and inhibited CD3ζ expression in CD8^+^ T lymphocytes. In addition, CD11b^+^CD14^−^ MDSC numbers were reduced in the patients who were responsive to treatment [14]. Feng and colleagues described the characteristics of CD11b^+^CD14^+^S100A9^+^ M-MDSCs in the peripheral blood and their clinical relevance in patients with advanced NSCLC. High levels of CD11b^+^CD14^+^S100A9^+^ M-MDSCs were associated with a poor response to cisplatin-based chemotherapy and predicted shortened progression-free survival (PFS) [15].

Huang et al. observed for the first time that both the absolute number and percentage of CD14^+^HLA-DR^−/low^ cells were increased in NSCLC patients with metastasis. Furthermore, both the percentage and absolute number of CD14^+^HLA-DR^−/low^ cells prior to therapy negatively correlated with the clinical response and PFS following cisplatin-based chemotherapy in advanced NSCLC patients, suggesting that increased levels of immunosuppressive CD14^+^HLA-DR^−/low^ cells might be associated with the poor prognosis of NSCLC. These suppressive cells upregulated the expression of gp91^phox^, an important component of the ROS-generating enzyme NADPH oxidase [17]. Subsequently, for small cell lung cancer (SCLC) patients, the frequency of CD14^+^HLA-DR^−/low^ MDSCs was also found to be negatively correlated with clinical outcomes [18]. Zhang et al. identified a new subset of MDSCs in the tumor microenvironment of NSCLC, B7-H3^+^CD14^+^HLA-DR^−/low^ MDSCs (B7-H3^+^ MDSCs). Their results suggested that the elevated frequency of this novel MDSC subpopulation correlated with a poor TNM stage and tumor metastasis and might be a predictor of poor recurrence-free survival (RFS) in patients with NSCLC. Further studies indicated that B7-H3^+^ MDSCs promoted cancer progression by producing IL-10 to induce Treg cells in the tumor microenvironment [19].

Scrimini et al. observed that the circulating levels of MDSCs (Lin^−^HLA-DR^−^CD33^+^CD11b^+^) and the serum concentration of Arg-1 were increased, and the surface expression of TCRζ in circulating lymphocytes was reduced to a similar extent among patients with LC, chronic obstructive pulmonary disease (COPD), or both diseases. These results suggested that tumor immunosurveillance might be impaired in COPD patients, which may contribute to the increased risk of LC reported in these patients [83].

Immunoglobulin-like transcript 3 (ILT3) is a receptor-containing immunoreceptor tyrosine-based inhibition motifs (ITIMs) that can be expressed on antigen-presenting cells and is an important regulator of DC tolerance [84, 85]. ILT3 exists in membrane-bound and soluble forms and can interact with a yet unidentified ligand on T cells to induce T cell anergy, Treg cells, or T suppressor cells [86, 87]. ILT3 is also expressed on MDSCs, and the ILT3^high^ fraction of PMN-MDSCs (CD11b^+^CD14^−^HLA-DR^−^CD33^+^CD15^+^) correlates with a relatively poor outcome in stage IV NSCLC patients [20]. Nonetheless, it is debatable whether ILT3 alone determines the immunosuppressive status of MDSCs.

Vetsika et al. used flow cytometry to study the percentages and correlations of MDSCs and different immune cells in the peripheral blood of 110 chemotherapy-naive NSCLC patients and healthy controls. They identified two new monocytic subpopulations (Lin^−^CD14^+^CD15^+^HLA-DR^−^ and Lin^−^CD14^+^CD15^−^HLA-DR^−^) and one granulocytic subpopulation (Lin^−^CD14^−^HLA-DR^−^) based on the expression of the markers CD15 and CD14 in the immature myeloid cell population (Lin^−^CD33^+^CD11b^+^HLA-DR^−^) in the peripheral blood. In this study, normal levels of Lin^−^CD14^+^CD15^+^HLA-DR^−^ M-MDSCs but not Lin^−^CD14^+^CD15^−^HLA-DR^−^ M-MDSCs at baseline were associated with improved patient PFS and OS by dramatically reducing the percentages of DCs and T helper cells via regulation of the expression of iNOS and ROS, respectively [22]. However, bevacizumab-based chemotherapy significantly reduces the levels of Lin^−^CD14^−^HLA-DR^−^ PMN-MDSCs [23].

Most studies focus only on the proportion of MDSCs in the peripheral blood of patients with LC. Yamauchi et al. also focused on changes in MDSCs in the tumor tissues of patients with resectable NSCLC [28]. A significant increase in the frequency of circulating M-MDSCs was observed in the NSCLC patients compared with healthy donors (HDs). Moreover, the frequencies of M-MDSCs and PMN-MDSCs were higher in the tumor tissue than in the peripheral blood of the same patients. This accumulation was associated with elevated concentrations of inflammatory mediators (CCL2, CCL3, CCL4, CCL5, IL-8, and CXCL10) involved in MDSC migration to and activation in the tumor microenvironment. An analysis of the MDSC immunosuppressive pattern showed increased programmed death-ligand 1 (PD-L1) expression on circulating cells from the patients compared with those from the HDs. Tumor PMN-MDSCs displayed higher PD-L1 expression levels than the same cell type in the peripheral blood. The frequency of CCR5 expression on circulating M-MDSCs was significantly higher in the patients than in the HDs. Clinical data analysis revealed negative correlations between RFS and the frequencies of PMN-MDSCs and CCR5^+^ M-MDSCs in the circulation but not in the tumors. Their findings suggest that the level of MDSCs in the peripheral blood but not in tumor tissue predicts recurrence following surgery [28].

### MDSCs are associated with chemotherapy resistance

As previously described, the ectonucleotidases CD39 and CD73 hydrolyze ATP or ADP into AMP and further yield adenosine in a highly coordinated enzymatic process [88, 89]. CD39 and CD73 are expressed by cancer cells and different immune cell populations and are responsible for the regulation of the balance between proinflammatory ATP and immunosuppressive adenosine in the tumor microenvironment [48, 49, 90, 91]. Li et al. demonstrated that CD39 and CD73 were expressed on MDSCs in the peripheral blood of patients with NSCLC and that CD39^+^CD73^+^ MDSCs had an immunosuppressive function. Further analysis revealed that the percentage of CD39^+^CD73^+^ MDSCs was decreased with increasing numbers of chemotherapy cycles in the stable disease (SD) and partial response (PR) groups, whereas there was a trend toward an increase in the percentage of CD39^+^CD73^+^ MDSCs in the progressive disease (PD) group, suggesting that the changes in CD39^+^CD73^+^ MDSC frequency in NSCLC patients may be sufficient for predicting chemotherapeutic response. The ectoenzymatic activities of CD39 and CD73 are required for MDSC-mediated suppressive and chemoprotective effects [21].

### The effect of radiotherapy on MDSCs

Navarro-Martin et al. performed immunophenotypic analysis of peripheral blood samples from seven patients with LC who were unfit for surgery and treated with stereotactic body radiotherapy (SBRT). The study provided novel evidence that SBRT had a systemic effect on the immune system. The observation that both CD33^+^CD11b^+^CD14^−^ PMN-MDSCs and CD33^+^CD11b^+^CD14^+^HLA-DR^−/low^ M-MDSCs exhibited decreases in immunosuppressive function is a salient finding of the study [27].

### MDSCs are associated with the efficacy of immune checkpoint inhibitor treatment

Based on the immunosuppressive function of MDSCs, these cells also play important roles in predicting the efficacy of anti-PD-1 therapy. It has been reported that the number of Lox-1^+^ PMN-MDSCs in nonresponders is significantly higher than that in responders after the 1st nivolumab treatment and that related factors involved in MDSC proliferation (CXCL2, CCL23, and CX3CL1) and recruitment (HMGB1) exhibit the same trend, suggesting that Lox-1^+^ PMN-MDSCs are a specific subset with immunosuppressive function in NSCLC patients. Patients with a relatively high ratio of Treg cells to Lox-1^+^ PMN-MDSCs in the blood after their 1st nivolumab treatment will have improved PFS. The ratio of Treg cells to Lox-1^+^ PMN-MDSCs in the blood after the 1st nivolumab treatment predicts early response in NSCLC patients and may provide clinicians with vital information on whether to continue or stop further anti-PD-1 therapy [24]. Limagne et al. studied a prospective cohort of metastatic NSCLC patients (*n* = 61) treated with second- or third-line nivolumab. They observed that no variable was significantly associated with PFS under anti-PD-1 treatment at baseline; however, a high level of Tim-3 expression in peripheral lymphoid cells and an accumulation of Lin^−^CD33^+^CD14^+^CD15^−^HLA-DR^−^ M-MDSCs shortly after the initiation of therapy were important factors that negatively affected the response to anti-PD-1 therapy. Moreover, Tim-3 expression on CD8^+^ T cells and galectin-9 expression on M-MDSCs blunted the secretion of IFNγ from CD8^+^ cells and were involved in the mechanisms of resistance to nivolumab [25]. A study of 53 patients with NSCLC receiving nivolumab showed that patients with baseline high value of PMN-MDSCs (≥ 6 cell/μl) had a significantly improved survival compared with those patients with a lower baseline data [26]. The results of MDSC’s effects for the immune checkpoint inhibitor treatment are inconsistent, and thus, multicenter, mechanistic studies are needed to obtain more definitive results.

### MDSCs in murine models of LC

Murine models are regularly used to study the relationship between MDSCs and LC. Chen et al. reported that the accumulation of MDSCs in aged mice and the expression of PD-L1 (B7-H1) on MDSCs were closely correlated with age. Inhibition of PD-L1 (B7-H1) significantly reactivated T cells and reduced the tumor progression mediated by MDSCs. In addition, IL-10 released in aged mice stimulated the expression of PD-L1 (B7-H1) on MDSCs [92]. Parallel increases in the level of galectin-3 and the number of MDSCs in vivo have been detected after cisplatin treatment [93]. Pulmonary **c**arbon nanotube (CNT) aspiration was shown to render the host susceptible to lung carcinoma formation in a murine metastasis/dissemination model. This effect was mediated by increased local and systemic accumulation of MDSCs [94]. Subsequent studies further revealed that in vivo exposure to CNT rendered lung MDSCs susceptible to tumor-induced expression of TGFβ, which was responsible for the inhibition of T cell activity by MDSCs [95]. Dajon et al., using a murine model of lung adenocarcinoma, found that stimulation of Toll-like receptor 7 (TLR7) expressed by adenocarcinoma cells modulated the immune infiltrate, leading to a significant expansion of MDSCs that was associated with increased secretion of CCL2 and GM-CSF in the tumor microenvironment. TLR7 stimulation had a prometastatic effect, while MDSC depletion drastically reduced the number of metastases. In line with the involvement of epithelial-mesenchymal transition (EMT) in the metastatic process, lung tumors expressing high levels of TLR7 have high expression of vimentin and low abundance of E-cadherin [96].

## Therapeutic MDSC-targeted strategies for inhibiting LC

As described above, a large body of evidence supports a close association between MDSC accumulation and clinical outcomes in LC patients. A recent meta-analysis of studies of various solid tumor patients showed that MDSCs were significantly associated with OS and PFS [97]. MDSCs are associated with anticancer therapies, including bevacizumab [23], cisplatin and other chemotherapeutic drugs [15, 21, 56], and recent studies have shown that MDSCs levels are associated with the response of patients to anti-PD-1 treatment [24, 25]. A variety of signaling pathways and cytokines are involved in the regulation of MDSCs. The interaction of all these factors constitutes a complex network control system that regulates the generation and function of MDSCs. To successfully implement anti-LC treatment, MDSCs, a major suppressive population in tumors, must be removed, so strategies targeting MDSCs are gradually being realized (Table 2). For example, the efficacy of therapeutic vaccination is increased when this approach is combined with MDSC depletion (anti-Gr1 antibody treatment) [9].

**Table 2:** MDSC-targeted strategies inhibit lung cancer progression

Refs.	Therapeutic strategy/compound	Targeted process	Tumor model	Implications
[98]	P53 vaccine and ATRA	Lin^−^HLA-DR^−^CD33^+^MDSCs depletion	Extensive stage SCLC patients	1. Enhancement p53-specific immune response 2. Better clinical response
[99]	Bevacizumab and EGFR TKI	Reduced the level of circulating S100A9 positive M-MDSCs	Patients with IV lung adenocarcinoma harboring an activating EGFR mutation	1. Improvement intracranial control rate and intracranial lesion TTP in patients with EGFR-mutant lung adenocarcinoma 2. Increased gene signatures associated with CD8 effector genes, Th1 chemokines, and NK cells.
[100]	Cabergoline	Reduced the accumulation of MDSCs	LLC1 murine model	1. Reduction angiogenesis 2. Inhibition lung cancer growth
[101]	Cimetidine	Reduced the accumulation of MDSCs	3LL murine model	1. Inhibition of tumor growth 2. Enhancement MDSCs apoptosis
[9]	Monoclonal anti-Gr1 or anti-Ly6G Abs	MDSC depletion	3LL murine model	1. Increased NK- and CD8+ T cell activity 2.Increased anti-angiogenic but reduced pro-angiog marker expression 3.Reduced 3LL lung metastases 4.Inhibition of tumor growth
[9]	BMA-OVA + anti-Gr1 Abs	MDSC depletion	3LL-OVA murine model	1. Tumor growth inhibition 2. Increased: splenic production of IFNγ and frequency of IFNγ producing CD4 and CD8 memory (CD44) and activation (CD69) marker expressing T cells
[102]	Gemcitabine + SOD mim	MDSC depletion	3LL murine model	1. Inhibition tumor growth 2. Enhances the quantity and quality of both effector and memory CD8^+^T cell responses. 3. Enhanced cytolytic CD8^+^ T cell response and further decreased Treg cell infiltration. 4. Improved long-term survival of mice bearing lung cancer 5. Thiol-dependent STAT-3 activation is enhanced in memory cells
[103]	IDO1 inhibitor	Reduced the percentages of F4/80^+^Gr1^int^CD11b^+^ MDSCs	Anti-PD-1-resistant cell line (344SQ-R) murine model	1. Inhibition suppresses tumor growth and lung metastases in anti-PD1 resistant tumors 2. Reduction both Kyn levels and Kyn:Trp 3. Reactivation CD8+ T cells 4. Reduction IDO1 expression of F4/80 + Gr1intCD11b + MDSCs and the percentage of IDO1^+^CD11b^+^ DCs in anti-PD1 resistant tumors.
[104]	Entinostat+ anti-PD-1 antibody	Reduced the immunosuppressive activity of MDSCs	3LL murine model	1. Enhanced anti-PD-1 immunotherapy 2. Decrease in the protein levels of FoxP3 in the circulating CD4^+^FoxP3^+^cell subtype 3. Increase in the CD8^+^ T - Treg cells ratio 4. Reduction of tumor infiltrating macrophages 5. Increase in MDSC associated trafficking/accumulation cytokines, anti-tumor chemokines, and cytokines
[105]	CCL2 antagonist+ anti-PD-1 antibody	Decreased MDSC recruitment	3LL murine model	1. Increased the survival time of tumor-bearing mice 2. Enhanced CD4+ and CD8+ T cell infiltration and activation
[106]	MEK Inhibitor (Trametinib) + either anti-PD-1 or anti-PD-L1 mAbs	Attenuation of Ly6Ghigh PMN-MDSCs	*p53*^*floxflox*^*;Kras*^*LSL-G12D/+*^*.R*_*0sa*_*26*^*LSL-Luciferase/LSL-Luciferase*^ (PKL) - transgenic lung cancer mouse model	1. Increased antitumor response and survival outcome 2. Increased of tumor-infiltrating CD8+ and CD4+ T cell 3. Suppressed tumor cell proliferation and lead to apoptosis of tumor cells
[107]	Carnosic acid	Decreased function and accumulation of MDSCs	3LL murine model	1. Enhanced the anti-growth effects of cisplatin on LLC xenografts and reduced the side effects of cisplatin. 2. Increased antitumor response 3. Enhanced cisplatin-induced tumor proliferation inhibition and apoptosis 4. Promoted CD8+ T cells-mediated antitumor immune response
[108]	Resveratrol	Decreased PMN-MDSC accumulation	3LL murine model	1. Increased antitumor response and survival outcome 2. Promoted the apoptosis of PMN-MDSCs, impair PMN-MDSCs immunosuppressive capacity 3. Boosted M-MDSCs maturation and differentiation
[109]	Curcumin	Decreased MDSC accumulation	3LL murine model	1. Increased antitumor response 2. Promoted the maturation and differentiation of MDSCs 3. Inhibited the expression level of Arg-1 and ROS 4. Decreased the level of IL-6

*ATRA* All-trans retinoic acid, *MEK* mitogen-activated protein kinase/extracellular signal-regulated kinase, *TTP* time to progress, *BMA-OVA* the vaccine consisted of bone marrow adherent cells (BMA) that had been pulsed with ovalbumen (OVA) protein, *SOD mim* superoxide dismutase mimetic

### All-trans retinoic acid

All-trans retinoic acid (ATRA) was historically the first therapeutic compound used to target MDSCs [110]. In previous studies, ATRA showed a direct effect on MDSCs, causing apoptosis in PMN-MDSCs and differentiating M-MDSCs into mature myeloid cells [110–112]. The effect of ATRA on MDSCs is linked to the upregulation of glutathione synthase and glutathione (GSH) expression in these cells. GSH dampens the level of ROS in MDSCs and thus promotes their differentiation [113]. Iclozan et al. found that for extensive SCLC, a combination of the P53 vaccine and ATRA reduced the proportion of Lin^−^HLA-DR^−^CD33^+^ MDSCs in the peripheral blood and increased the vaccine-induced immune response to nearly 42% [98].

### Bevacizumab

In vivo and in vitro analyses have shown that VEGF released from the tumor microenvironment stimulates the mobilization of MDSCs from the bone marrow to the peripheral blood, thus leading to increased MDSC levels in the peripheral blood. In addition, VEGF contributes to the selective accumulation of MDSCs within tumor sites [114]. Therefore, VEGF may not only promote tumor growth but also participate in a suppressive process that limits antitumor immunity [115]. Bevacizumab has been demonstrated to provide a survival benefit when used in combination with platinum-based doublet chemotherapy in patients with nonsquamous NSCLC. For NSCLC patients, three cycles of bevacizumab-containing regimens compared with non-bevacizumab-based regimens significantly reduced the percentage of PMN-MDSCs [23]. The combination of bevacizumab and an EGFR tyrosine kinase inhibitor (TKI) robustly improved the intracranial control rate and intracranial lesion time to progression (TTP) in patients with EGFR-mutant lung adenocarcinoma, possibly by reducing the level of circulating S100A9-positive M-MDSCs, along with enhancing gene signatures associated with CD8^+^ T cell effector genes, Th1 chemokines, and NK cells [99].

### Dopamine D2 receptor agonists

Studies have shown that dopamine (DA) stimulates the endocytosis of VEGFR-2 via dopamine D2 receptor (D2R), thereby preventing angiogenesis by inhibiting VEGF binding, receptor phosphorylation, and subsequent downstream signaling [116]. Subsequent studies by numerous investigators have clearly demonstrated that this strategy can be successfully applied to various diseases, including cancer [117, 118]. Hoeppner et al. demonstrated that D2R agonists (Cabergoline) inhibited NSCLC tumor progression in orthotopic lung tumor models through two mechanisms: a reduction in tumor angiogenesis in lung endothelial cells and abrogation of tumor-infiltrating MDSCs [100].

### Cimetidine

Cimetidine, a histamine type-2 receptor antagonist, is known to inhibit the growth of several tumors in humans and animals [101, 119, 120]. Zheng et al. found that cimetidine significantly inhibited tumor growth in 3LL lung tumor mouse models and reduced the accumulation of MDSCs in the spleen, blood, and tumor tissue. The underlying mechanisms were as follows: first, cimetidine affected the expression of Fas/FasL signaling in MDSCs; then, cimetidine induced apoptosis in MDSCs in a caspase-dependent manner [101].

### Superoxide dismutase mimetic

MDSCs produce ROS-associated free radicals and immunoregulatory cytokines to inhibit host CD4^+^ and CD8^+^ T cell responses, thereby promoting tumor progression and metastasis [7, 121]. In a mouse model of homologous LC, the combination of a superoxide dismutase mimetic (SOD mim) targeting ROS and gemcitabine not only impaired MDSC function and induced an effective memory T cell response against lung tumors but also reduced tumor growth and resulted in long-term immunity and survival. The cellular redox-mediated activation of STAT-3 and regulation of central memory and effector memory CD8^+^ T cell metabolism contributed to long-term immunity. Furthermore, adoptive transfer experiments demonstrated that inhibiting ROS produced by MDSCs and MDSCs enhanced the persistence of memory CD8^+^ T cells and their strong activity in response to re-exposure to tumor antigens during the relapse phase [102].

### Carnosic acid

Liu et al. found that the drug carnosic acid, which has antioxidant and antibacterial properties, could inhibit the function of MDSCs in tumor models and enhance the anti-LC activity of cisplatin. The mechanism might involve decreased expression of Arg-1, iNOS2, and MMP9. In addition, in their experiments, CD8^+^ T cells were found to be present mainly in tumor tissues after treatment with carnosic acid. At the same time, the mRNA expression of Perforin, Granzyme B, and FasL was boosted, which might indicate that carnosic acid promoted the functions of CD8^+^ T cells and contributed to tumor suppression together with cisplatin. Therefore, carnosic acid may be a new candidate for LC combination therapy [107].

### Resveratrol

Resveratrol (RSV) is a pleiotropic phytochemical found in peanuts and grapes that has been indicated to provide a wide range of health benefits, such as reducing oxidative, inflammatory, and apoptotic signals [122]—protecting against neurological decline [123], improving cardiovascular health [124], ameliorating diabetes [125], and preventing cancer [126]. The anticancer properties of RSV act through diverse molecular mechanisms that have been investigated in a plethora of cellular and animal models [127, 128]. Zhao et al. demonstrated for the first time that RSV could reduce the accumulation of MDSCs in vivo and in vitro while delaying the progression of cancer in LLC tumor-bearing murine models. In vitro results indicated that RSV attenuated PMN-MDSC expansion by selectively inducing apoptosis and the differentiation of M-MDSCs into Mø and reducing the recruitment of PMN-MDSCs [108]. These results indicate that RSV should be considered a modulator of MDSC suppressive function and that RSV is a novel potentiator for tumor immunotherapy.

### Curcumin

Curcumin, a yellow substance derived from turmeric that belongs to the polyphenol superfamily, is a highly pleiotropic molecule. Curcumin combined with vaccination leads to decreased numbers of MDSCs and Treg cells, decreased levels of IL-6 and an elevation in the CD8^+^ T cell population in an advanced melanoma model [129]. In LLC tumor models, a curcumin-based treatment strategy was shown to reduce the frequency and absolute number of MDSCs in the spleen and tumor tissues, significantly reduce IL-6 levels in tumor tissues and the serum, weaken the Arg-1 expression level and ROS production in MDSCs purified from tumor tissue in vivo, and promote the expression of the maturation markers F4/80, MHCII, CD11c, and CD80 on MDSCs in tumors. Since IL-6 could promote the expansion and suppressive function of MDSCs [130], it was suggested that curcumin might inhibit the accumulation and suppressive function of MDSCs partly by reducing the level of IL-6 in tumor-bearing mice [109].

### Increased immune checkpoint inhibitor efficacy

In addition to the abovedescribed therapies, immune checkpoint inhibition is a novel treatment that is being studied extensively in LC. Many of the studies have concluded that MDSCs are associated with resistance to anti-PD-1 therapy [24, 25] and that there is an elevated level of PD-L1 expression on MDSCs [28]; thus, targeting MDSCs has the potential to increase anti-PD-1 efficacy. Studies by Li et al. provided preclinical evidence that anti-PD1 resistance in LC was associated with overexpression of IDO1 in F4/80^+^Gr1^int^CD11b^+^ MDSCs. Treatment with an IDO1 inhibitor (INCB023843) reduced IDO1 expression and blocked the accumulation of F4/80^+^Gr1^int^CD11b^+^ MDSCs, resulting in reactivation of the antitumor immune response. Thus, IDO1 inhibition represents another immunotherapeutic strategy to overcome the immunosuppressive TIME in anti-PD1 therapy-resistant tumors [103]. Entinostat is an oral, class I-specific histone deacetylase inhibitor (HDACi) that disrupts the dynamic interactions between the tumor microenvironment and host immune surveillance [131]. Orillion and colleagues used entinostat in combination with PD-1 inhibitors to reduce tumor growth and increase survival in subcutaneous LC models. The underlying mechanisms of this combination might be significant decreases in the arginase-1, iNOS, and COX-2 levels and changes in the cytokines/chemokines released in vivo with a shift from an immunosuppressive microenvironment to an immunostimulatory microenvironment [104]. Wang et al. showed that MDSC subsets had a positive correlation with CCL2. In addition, combination treatment with a CCL2 antagonist and anti-PD1 antibody enhanced CD4^+^ and CD8^+^ T cell infiltration, as well as the production of IFNγ, and increased the survival time of tumor-bearing mice [105]. *KRAS* mutations, known as oncogenic driver mutations, have been detected in 20~40% of adenocarcinomas and in 3~6% of squamous cell carcinomas [132]. Targeting the downstream target of *KRAS* signaling, mitogen-activated protein kinase/extracellular signal-regulated kinase (MEK) in *KRAS*-mutated NSCLC has been reported to be largely ineffective [133, 134]. However, the combination of a reduced dose of a MEK inhibitor and an immune checkpoint inhibitor for treatment of a Kras/p53-driven lung tumor was shown to significantly prevent tumor growth and extend survival in preclinical models. In addition, the mechanisms may be as follows: (1) enhancing T cell infiltration both in the tumor microenvironment and inside the tumor bed, (2) decreasing the number of PMN-MDSCs in the tumor, and (3) suppressing tumor cell proliferation and leading to apoptosis in tumor cells [106].

### CD33 antibody

The anti-MDSC methods described above are either based on mouse models or do not have a selective depleting effect on human MDSCs, but rather extensive immune regulation. Therefore, we need to explore the intentional marker for human MDSCs as a better way to consume MDSCs. CD33, a defining marker of MDSCs (CD33^+^HLA-DR^−^Lin^−^), is highly expressed in myelodysplastic syndrome (MDS) MDSCs. Eksioglu et al. took advantage of the novel, fully human CD33 antibody BI 836858, which is Fc-engineered for increased binding to Fcγ receptor IIIa (FcγRIIIa), and observed that BI 836858 can reduce MDS MDSCs through antibody-dependent cellular cytotoxicity (ADCC) and that this effect is correlated with increases in granule mobilization and cell death. BI 836858 can also reduce both ROS and the levels of double-strand breaks and adducts [135]. One of the main challenges in targeting human MDSCs is their heterogeneity, as well as differences in immune phenotypes and intracellular suppression mechanisms between the same patient (blood and tumor) and across different types of cancer. Fultang et al. identified CD33 as a therapeutic target on peripheral and infiltrating MDSCs across cancer subtypes by RNA-sequencing and flow cytometry. Gemtuzumab ozogamicin (GO), a humanized anti-CD33 IgG4 mAb conjugated to a cytotoxic agent *N*-acetyl gamma calicheamicin via an acid-labile hydrazone linker [136], has been successfully used as a treatment for acute myeloid leukemia [137]. The argeting of MDSCs with GO leads to GO internalization, increased p-ATM, and MDSC cell death. Anti-GD2-/mesothelin-/EGFRvIII-CAR-T cell activity is enhanced in combination with the anti-MDSC effects of GO [138]. This research suggests that, for patients with high levels of MDSCs, immune “cold” tumors may be transferred into immune “hot” tumors via GO therapy to improve the efficacy of chemotherapeutic and/or immunotherapeutic regimens.

Both of the above studies consider that CD33 is a specific marker for human MDSCs and has a significant effect on depleting MDSCs. These two studies provide a better support for targeting the immunosuppressive TIME of LC patients.

## Conclusions

Tumor cells develop a variety of mechanisms to evade the immune system and undergo progression. One of the key mechanisms is the establishment of an immunosuppressive TIME, in which MDSCs play a crucial role. There is increasing evidence that MDSCs are involved in the development of LC and may be used to predict the efficacy of immune checkpoint blockade treatment, but there are still a wide range of unknown mechanisms and interactions that require further research within this topic. Based on the description herein, various preclinical and clinical studies have shown the beneficial effects of altering the function and biology of MDSCs. These findings suggest that targeting MDSCs may be a promising strategy for use with existing immunotherapeutic strategies, such as boosting the immune system by vaccination or immune checkpoint inhibition. Using these strategies to treat LC may produce more breakthroughs that overcome current treatment limitations. However, these findings still require a more solid research foundation before clinical translation. First, unlike other immunosuppressive cells (e.g., Treg cells and TAMs), MDSCs do not have a uniform molecular phenotype. Second, the results for the relationships between different MDSC subtypes and the prognosis of LC are not consistent. For example, some studies suggest that elevated PMN-MDSC numbers are an indicator of a poor prognosis [20, 24], while other studies show that M-MDSCs have a better prognostic value than PMN-MDSCs [15, 22, 25]. Third, although MDSCs can be detected in the peripheral blood and tumor tissue, it is difficult to use the level of MDSCs in the peripheral blood to represent the distribution in tumor tissues, and the functional level is not predictable. Therefore, more in-depth explorations of the mechanisms of MDSCs in tumor tissues are still needed for LC patients. Finally, reasonable pharmacological data related to targeted MDSC therapy remain unresolved. It is hoped that this topic will be analyzed using a comprehensive epidemiological model to fully understand the value of collecting MDSC measurements for patient outcomes and to reduce the number of potential modifiable factors for MDSC accumulation in LC patients. Additional in-depth institutional studies will provide a more reliable basis for MDSC targeting either alone or in combination with chemotherapeutic and/or immunotherapeutic regimens.

## Data Availability

The dataset supporting the conclusions of this article is included within the article.

## Abbreviations

Arg-1: Arginase 1
CARD9: Caspase recruitment domain-containing protein 9
CDK4: Cyclin-dependent kinase 4
DCs: Dendritic cells
EMT: Epithelial-mesenchymal transition
HDAC: Endogenous inhibitor of histone deacetylases
HIF-1α: Hypoxia-inducible factor-1α
iNOS: Inducible nitric oxide synthase
LC: Lung cancer
MDSCs: Myeloid-derived suppressor cells
M-MDSCs: Monocytic-MDSCs
Mø: Macrophages
NF-κB: Nuclear factor-κB
NOS2: Nitric oxide synthase 2
NSCLC: Non-small cell lung cancer
PMN-MDSCs: Polymorphonuclear-MDSCs
ROS: Reactive oxygen species
SCLC: Small cell lung cancer
STAT: Signal transducer and activator of transcription
TAMs: Tumor-associated macrophages
TF: Tissue factor
TIME: Tumor immune microenvironment
TLR7: Toll-like receptor 7
Treg: Regulatory T
VEGF: Vascular endothelial growth factor
